# Inferring evolution of gene duplicates using probabilistic models and nonparametric belief propagation

**DOI:** 10.1186/1471-2164-14-S1-S15

**Published:** 2013-01-21

**Authors:** Jia Zeng, Sridhar Hannenhalli

**Affiliations:** 1 School of Computer Science and Technology, Soochow University, Suzhou 215006, China; 2 Shanghai Key Laboratory of Intelligent Information Processing, China; 3 Center for Bioinformatics and Computational Biology, University of Maryland, College Park, MD 20742, USA

## Abstract

**Background:**

Gene duplication, followed by functional evolution of duplicate genes, is a primary engine of evolutionary innovation. In turn, gene expression evolution is a critical component of overall functional evolution of paralogs. Inferring evolutionary history of gene expression among paralogs is therefore a problem of considerable interest. It also represents significant challenges. The standard approaches of evolutionary reconstruction assume that at an internal node of the duplication tree, the two duplicates evolve independently. However, because of various selection pressures functional evolution of the two paralogs may be coupled. The coupling of paralog evolution corresponds to three major fates of gene duplicates: subfunctionalization (SF), conserved function (CF) or neofunctionalization (NF). Quantitative analysis of these fates is of great interest and clearly influences evolutionary inference of expression. These two interrelated problems of inferring gene expression and evolutionary fates of gene duplicates have not been studied together previously and motivate the present study.

**Results:**

Here we propose a novel probabilistic framework and algorithm to simultaneously infer (i) ancestral gene expression and (ii) the likely fate (SF, NF, CF) at each duplication event during the evolution of gene family. Using tissue-specific gene expression data, we develop a nonparametric belief propagation (NBP) algorithm to predict the ancestral expression level as a proxy for function, and describe a novel probabilistic model that relates the predicted and known expression levels to the possible evolutionary fates. We validate our model using simulation and then apply it to a genome-wide set of gene duplicates in human.

**Conclusions:**

Our results suggest that SF tends to be more frequent at the earlier stage of gene family expansion, while NF occurs more frequently later on.

## Background

Gene duplication is one of the major drivers for functional evolution, as it allows one or both duplicates to diversify their functions relative to the ancestral function [1]. Fixed gene duplicates that evade pseudogenization and extinction follow one of the three major fates: subfunctionalization (SF), conserved function (CF) or neofunctionalization (NF) [1,2]. In the case of SF, both gene duplicates accumulate random mutations such that each duplicate retains a complementary subset of the ancestral function and the overall ancestral function is performed jointly by the two gene copies. The Duplication-Degeneration-Complementation (DDC) model is one of the explanatory models of SF [3]. Under CF, both copies retain the ancestral function, consequently "doubling" the ancestral function, i.e., increase dosage of the gene product. In the case of NF, one duplicate retains the ancestral function and the other duplicate, under relaxed selective pressure, acquires novel functions. The relative propensity of the three evolutionary fates and their underlying mechanisms are of considerable research interests [3,4].

Investigating functional divergence of paralogs is challenging at many levels. For one, what constitutes a "function" itself is not straightforward. In this context, several proxies have been used to represent gene function including protein-protein interactions (PPI) [5-7], regulatory networks [8], fitness effect [9], metabolic networks [10,11], genetic interactions [12], and gene expression patterns [13-16]. Each of these alternatives necessitates a different model to quantify the three fates in the functional evolution of paralogs. For instance, if PPI is used, the function of a gene *X *is {*Y*: *PPI*(*X, Y*)}, where *PPI*(*X, Y*) indicates physical interactions between proteins *X *and *Y*. Shared interactions are then used to quantify shared functions between two genes. These previous approaches have a few limitations, motivating our current work. First, analogous to "interacting proteins", the "expression domain" (i.e., the tissues where a gene is expressed) has been used as a surrogate for function [6]. However, the expression level of a gene in a specific tissue is also important for its function, and this aspect of gene function has not been used to investigate evolutionary fates of paralogs, partly because of noisy expression data. A second limitation in previous investigations of evolutionary fates of paralogs relates to inference of the ancestral function. Previous works [5-7,17] quantify the extent of SF and NF by investigating the functional overlap between paralogs as it relates to inferred age of gene duplication event, but not based on explicit inference of ancestral function. For instance, [17] has proposed various models of expression evolution to conclude that expression tends to diverge soon after duplication and expression change is decoupled from genetic distance. However, in [17], the ancestral expression is set to equal-weighted average of descendent expression values. Such approaches assume that the duplicate genes diversify independently, which is not the case as the postulated evolutionary fates of duplicates--SF, NF, CF--implicitly assume coupled evolution. While the inference of evolutionary fates requires inference of ancestral expression, inferring the latter depends on evolutionary fates at internal nodes. The third limitation of previous works is that they do not integrate these two interrelated evolutionary inference problems. Thus the primary contribution of our work is to propose a novel probabilistic integrative framework for the dual inference problem. In particular, to address the three limitations of the previous works, the methodological contributions of our work are: (1) use of tissue-specific expression level as a surrogate for gene function, (2) accurate inference of ancestral gene expression accounting for SF, NF, CF, and (3) development of a probabilistic model to infer SF, CF, NF based on predicted and known expression levels.

We propose a new description of SF, CF and NF using tissue-specific gene expression level as a surrogate for gene function. In our framework, CF implies that the two duplicates retain the ancestral expression level. SF implies that the duplicates have reduced levels of expression such that the sum of their expression levels is equal to that of the ancestor. NF implies that one of the duplicates retains the expression level while the other has increased expression such that the sum of expression levels of duplicates is much greater than twice that of the ancestor. A key step in our proposed approach is to predict the ancestral gene expression level based on the phylogenetic tree of the paralogs and known expression levels at leaves. We modify the nonparametric belief propagation (NBP) [18] algorithm for this task. NBP is a graphical model-based tracking algorithm widely used in human motion tracking problems such as the gesture recognition. In that context, various component gestures are interpreted in the context of other component gestures (a raised arm while not moving may not mean the same thing as a raised arm while running). We imagine that the gene expression level evolves along the phylogenetic tree from internal nodes to leaf nodes, and such evolutionary "movement" can be tracked by the NBP algorithm.

We propose a model that probabilistically classifies each gene duplication event, including an ancestral node and the two children nodes, into three possible evolutionary fates, based on the predicted (or known) gene expression levels at three nodes. This model appropriately incorporates the unavoidable ambiguity in interpretation of SF, CF and NF, and thus provides a robust quantitative inference of relative propensities of the three evolutionary fates in gene duplication. Application of our tool to human tissue-specific gene expression data including 783 paralogs grouped into 261 three-paralog families and 592 paralogs grouped into 148 four-paralog families in 79 tissues reveals that SF tends to occur more frequently in older gene duplication events, while CF and NF are predominant among younger gene duplicates.

The following is organized as follows. In Methods, we present a new definition of SF, CF and NF based on tissue-specific expression levels. Using this definition, we propose a novel probabilistic model to quantify the three evolutionary fates including SF, CF and NF based on the predicted ancestral function from the phylogenetic tree using the nonparametric belief propagation algorithm. In Results, we present extensive experimental results. In Conclusions and Discussions, we discuss the intrinsic relations between our findings and other evidence of gene functional divergence, and draw conclusions and envision future work.

## Methods

**Figure 1:**
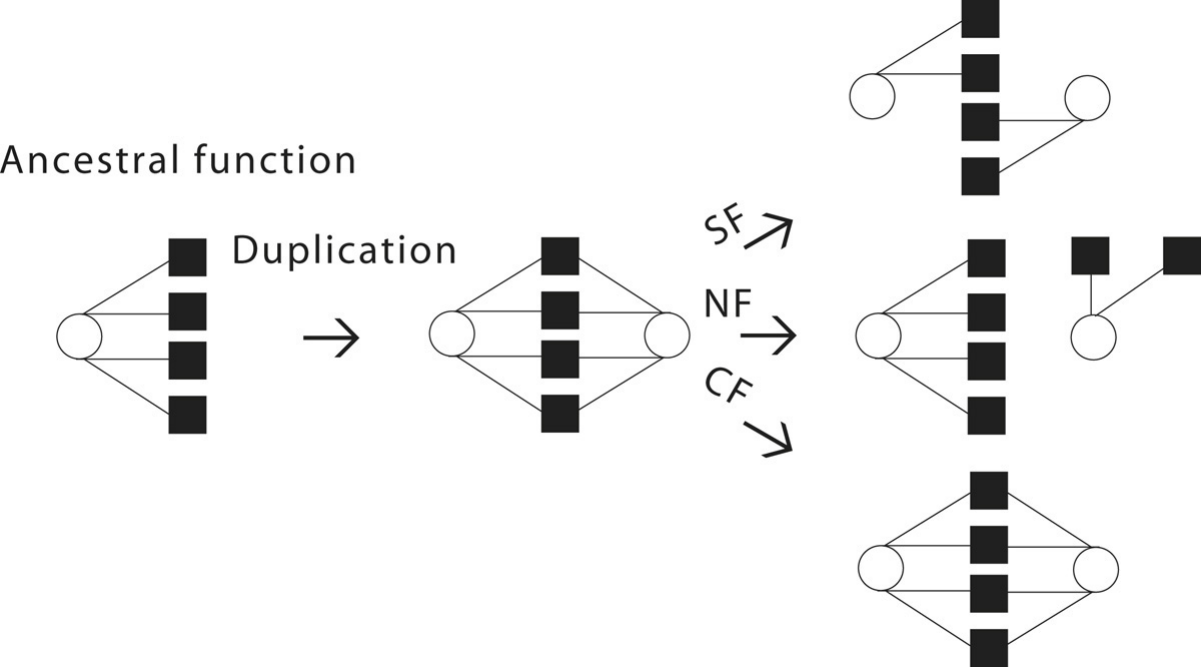
**Three fates of functional evolution **[6]**based on tissue-specific gene expression patterns, where circles denote genes, squares denote tissues, and edges denote that genes are expressed in tissues**. Immediately after gene duplication, both duplicates retain the same set of ancestral functions denoted by the same set of edges. Under pure SF a complementary set of ancestral edges are retained by each duplicate. Under pure CF both duplicates retain the same set of ancestral edges. Under pure NF one duplicate retains the ancestral edges while the other duplicate develops new edges with other tissues.

We first summarize the most relevant previous work that motivates our current work. Besides PPI, tissue-specific gene expression patterns offer another important aspect of gene function. Using the conventional cutoff values, as illustrated in Figure 1, continuous expression levels are transformed to binary patterns {0, 1} for unexpressed and expressed genes in 25 independent tissues [6]. Thus, gene function can be represented by a binary vector of length 25, for example, [0, 1, ..., 0, 1, 0]^T^, where 0 or 1 denotes that the gene is unexpressed or expressed in a certain tissue.

The average number of functions of singleton genes is used as the average number of ancestral functions, because singleton genes have no duplicates in the genome [6]. Under this scenario, SF would result in maintenance of total function while NF would result in gradual increase in function over time. While pure SF and NF are rare, a large fraction of gene duplicates undergo rapid SF followed by prolonged period of NF referred to as the sub-neo-functionalization (SNF) model [6]. However, CF was excluded from the SNF model. This is because CF would predict the average number of functions per gene duplicate to be equal to the average number of functions per singleton gene, which is inconsistent with the observed data.

The above approach has a few shortcomings. First, pure SF, CF and NF might have been deemed rare partly because of the description of SF, CF and NF based on multiple tissues. Taking three tissues as an illustrative example, SF posits complementary functions of paralogs, such as [1, 0, 1] and [0, 1, 0]. While it may be easy to find a pure SF pattern in three tissues, with larger number of tissues as in [6], even a low level of noise in gene expression data will make it highly improbable to find exact complementary expression patterns. The same reasoning applies to CF and NF. Second, the inferences may change substantially if additional tissues are included in the analysis. Finally, SF, CF and NF can be best inferred only if we know (or predict) ancestral function (expression in this case). For example, consider two paralogs having functions [1, 0, 1] and [0, 1, 0]. If their ancestral function is [1, 1, 1], SF should be inferred. But if their ancestral function is [1, 0, 1], NF is more likely. In this regard, while the evolutionary fate at a duplication node determines the expression levels of the descendent nodes, the previous approaches to estimating the ancestral gene expression level [17] are oblivious of evolutionary fates. To address these problems, next we propose a new description of function based on a single tissue gene expression level.

### A new description of functions

In this initial treatment of the problem, we have implemented a simple description of SF, CF and NF based on the single tissue-specific gene expression level. For example, given the liver-specific gene expression level, we define SF, CF and NF as

(1) SF:x=a+b,

(2) CF:x=a=b,

(3) NF:x=min(a,b),

where *a *and *b *are the expression levels of two gene duplicates, while *x *is the expression level of their ancestor. Eq. (1) indicates that the sum of gene expression levels of two gene duplicates is equal to their ancestral gene expression level. Such view of SF has also been discussed in [13], where the two gene duplicates produce the same amount of expression levels, and thus serve the same function as their ancestor. Eq. (2) indicates that both duplicates retain the ancestral expression level, thereby increasing the overall dosage of the gene product. Eq. (3) is consistent with one duplicate retaining the ancestral expression level, while the other gaining in expression level, analogous to a gain of new function. However, NF does include the possibility of both duplicates having increased expression level relative to the ancestor. For convenience, we normalize the three expression levels by their maximum value as follows,

(4) $$x=\frac{x}{\text{max}\left(x,a,b\right)},a=\frac{a}{\text{max}\left(x,a,b\right)},b=\frac{b}{\text{max}\left(x,a,b\right)}.$$

After normalization, we obtain *x, a, b *∈ 0[1] for the following probabilistic modeling and classification.

**Figure 2:**
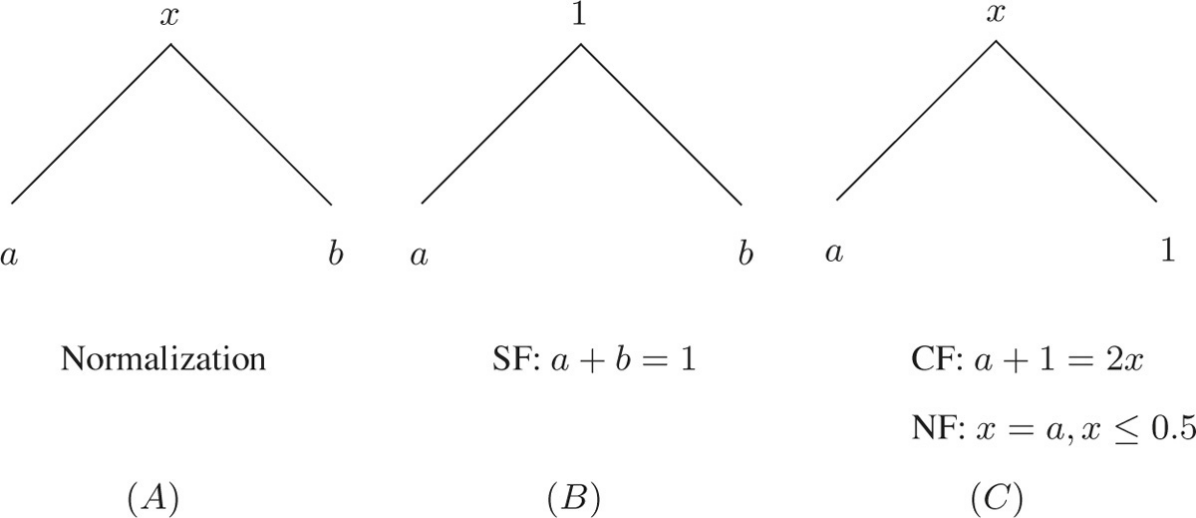
**There are two cases (*B*) and (*C*) after normalizing by the maximum value max(*x, a, b*) in (*A*)**. In (*B*), the maximum value is *x *= 1 after normalization. SF is defined by *a *+ *b *= 1. In (*C*), the maximum value is *b *= 1 after normalization. CF and NF are defined by 1 + *a *= 2*x *and *x *= *a, x *≤ 0.5, respectively.

Figure 2A shows the normalized expression levels {*x, a, b*}. After normalization, we obtain two cases in Figure 2B and 2C. Figure 2B shows that the ancestor has the maximum value *x *= 1. In this case, SF is defined as

(5) a+b=1.

This definition is the same as (1). Figure 2C shows that one of the duplicates has the maximum value *b *= 1. In this case, CF is defined as

(6) a+1=2x.

This definition is slightly different from (2), which requires *x *= *a *= *b*. In real gene expression data, the exact *x *= *a *= *b *is rare, therefore we relax this pattern by *a *+ *b *= 2*x*, which means that the total function of two duplicates is equal to the twice the ancestral function. In Figure 2C, because *a *is relatively smaller than *b*, NF is defined as

(7) x=a,x≤0.5,

This definition is similar to (3) but with the constraint *x *≤ 0.5. Under NF, when one duplicate *a *retains the ancestral expression level *x*, we assume that the other duplicate *b *has at least twice the expression level of the ancestor *x*. This constraint aims to distinguish NF from CF when *b *is very close to *x*.

### A probabilistic model for classification

**Figure 3:**
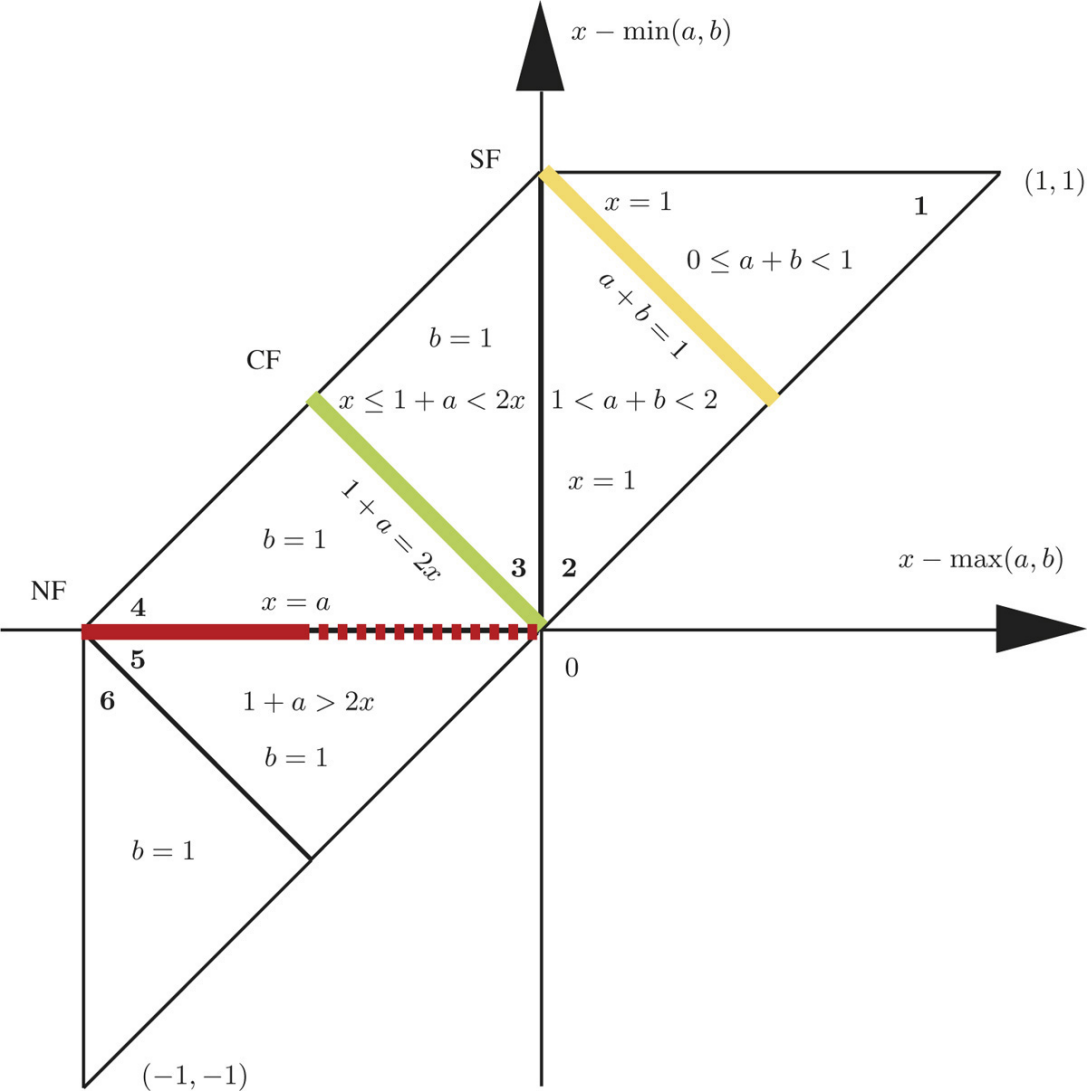
**The plane for the feature point {*x *- max(*a, b*), *x *- min(*a, b*)}**. All feature points are located within the trapezoid region composed of six triangular regions. Regions **1 **and **2 **correspond to *x *= 1 in Fig. 2B. Regions **3 **to **6 **correspond to *b *= 1 in Fig. 2C. The yellow line represents *a *+ *b *= 1 for SF in Eq. (5). The green line represents *a *+ 1 = 2*x *for CF in Eq. (6). The solid red line represents *x *= *a, x *≤ 0.5 for NF in Eq. (7). Each feature point can be classified as SF, CF, or NF fate by its distance to the three lines.

After normalization, we aim to classify each gene duplication event with feature point {*x, a, b*} into SF, CF and NF evolutionary fates. Without loss of information (because max(*x, a, b*) = 1), we transform the feature point {*x, a, b*} into {*x *- max(*a, b*), *x *- min(*a, b*)}, which can be illustrated by the Cartesian coordinate system in the plane as shown in Figure 3. We see that all feature points fall within the trapezoid region composed of six triangular regions. Figure 2B corresponds to triangular regions **1 **and **2 **with *x *= 1, while Figure 2C corresponds to triangular regions **3 **to **6 **with *b *= 1. The feature points located on the yellow line follow the SF definition *a *+ *b *= 1 in Eq. (5). The feature points located on the green line follow the CF definition *a *+ *b *= 2*x *in Eq. (6). The feature points located on the solid red line follow the NF definition *x *= *a, x *≤ 0.5 in Eq. (7). Although both solid and dashed red lines follow the definition for NF in Eq. (3), the dashed red line overlaps with the green line at origin point. So, we add the constraint *x *≤ 0.5 to distinguish NF from CF. All other feature points except those on the three lines can be viewed as SF, CF and NF with different probabilities.

The points in region **1 **satisfy the equation 0 ≤ *a *+ *b *< 1, which means that the total function of two gene duplicates is less than their ancestral function. The extreme case is the point (1, 1), which means that both duplicates lose their ancestral function, referred to as *gene expression loss*. The points in regions **2 **and **3 **follow equations 1 <*a *+ *b *< 2 and *x *≤ 1 + *a *< 2*x*, which means that the total function of two duplicates is higher than their ancestral function but less than twice their ancestral function. The points in regions **4 **to **6 **satisfy the equation 1 + *a *≥ 2*x*, which means that the total function of two duplicates is higher than twice of their ancestral function. The extreme case is the point (-1, -1), which means that both duplicates gain new functions, referred to as *gene expression gain*. Notice that gene loss can be viewed as a special case of SF, where both duplicates degenerate and lose the ancestral function. Gene expression gain can be viewed as a special case of NF, where both duplicates evolve novel functions. So, we do not consider these two specific cases in the following classification.

The colored lines represent the *pure *cases of SF, CF and NF. But most points fall in the intervening spaces, thus motivating a probabilistic method to classify these feature points. For simplicity, we classify the feature point by its distance to three lines. Thus, the probability that a feature point belongs to SF is

(8) $$p_{\text{SF}}=\frac{d_{\text{CF}}d_{\text{NF}}}{d_{\text{CF}}d_{\text{NF}}+d_{\text{SF}}d_{\text{NF}}+d_{\text{CF}}d_{\text{SF}}},$$

where *d*_SF_, *d*_CF _and *d*_NF _are distances between the feature point and the yellow, green and red lines, respectively. The probabilities of CF and NF can be calculated similarly, and it follows that

(9) $$p_{\text{SF}}+p_{\text{CF}}+p_{\text{NF}}=1.$$

If points are uniformly distributed in the trapezoid region, then more points will be closer to the red line, i.e., NF, just by chance.

### Predict ancestral expression levels

**Figure 4:**
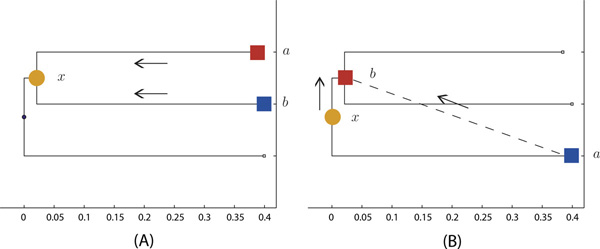
**(A) The forward particle passing procedure**. The particles of two duplicates *a *and *b *are passed to infer their ancestral particle *x*. (B) The backward particle passing procedure. The ancestral particle *x *and particle of one duplicate *a *are passed to infer the other duplicate *b*.

We have to infer the ancestral gene expression level to classify feature points {*x, a, b*} into SF, CF and NF fates. Based on the sequence similarity of paralogs, we first construct a phylogenetic tree composed of internal and leaf nodes denoted by circles and squares in Figure 4. The branch length denotes the number of synonymous substitutions per synonymous site (*dS*), which serves as a surrogate of time since duplication. Larger *dS *corresponds to longer evolutionary time. Given the gene expression levels at leaf nodes, we aim to infer the expression levels at internal nodes.

A typical approach to predicting the ancestral state (gene expression in our case) (such as [19]) attempts to explain the evolution by the minimum possible change, which may bias the inference of evolutionary fate of gene duplicates towards CF. However, a different fate at an internal node would yield a different expectation for the gene expression of the duplicates. We therefore modify the Nonparametric Belief Propagation (NBP) [18] to infer ancestral gene expression levels. NBP allows us to incorporate evolutionary fates (SF, CF, NF) at each internal node to infer ancestral gene expression level. We assume a uniform prior on SF, CF and NF.

For each internal node, the gene expression level *x *follows a three-component Gaussian mixture model (GMM) [20] corresponding to the three evolutionary fates,

(10) $$p\left(x\right)=\overset{3}{\underset{i=1}{\sum }}w_{i}N\left(x|\mu_{i},\sigma_{i}\right),$$

where *w_i _*is the mixing weight and $N\left(x|\mu_{i},\sigma_{i}\right)$ is the Gaussian distribution with the mean *μ_i _*and the standard deviation *σ_i_*. Each NBP iteration uses an efficient sampling procedure to update the GMM parameters based on the iterative Expectation-Maximization (EM) algorithm [20]. The EM algorithm is a widely used method for estimating parameters of finite mixture models like GMM [20]. The random samples drawn from GMMs are referred to as *particles*. The NBP algorithm infers particles at internal nodes by the forward-backward particle passing procedure as follows. The forward procedure passes possible gene expression levels of two duplicates to their ancestor, while the backward procedure passes possible expression levels of the ncestor and one of the duplicates to the other duplicate. Both procedures ensure that each internal node can obtain the complete information from its connected nodes.

#### Forward-backward particle passing

Figure 4A shows the forward particle passing procedure, which passes particles *a *and *b *of two duplicates to calculate the particle *x *of their ancestor according to SF, CF and NF fates:

(11) SF:x=a+b,

(12) $$\text{CF}:\;x=\frac{l_{b}*a+l_{a}*b}{l_{a}+l_{b}},$$

(13) NF:x=min(a,b),

where *l_a _*is the branch length from *x *to *a*, and *l_b _*is the branch length from *x *to *b*. The equations, except (12), are the same as Eqs. (1) to (3). Notice that (12) also satisfies the CF definition (2) but uses branch lengths as weights, thus using the minimum evolution as the criterion [19], which is the basis for CF. At each NBP iteration, we sample *M *particles corresponding to each of the three fates for *x *resulting in a total of 3*M *particles based on Eqs. (11) to (13).

Figure 4B shows the backward particle passing procedure, which passes the particles of ancestor *x *and particles of one duplicate *a *to the other duplicate *b*. According to SF, CF and NF fates, we obtain

(14) SF:x>a,b=x-a,

(15) $$\text{CF}:\;b=\frac{\left(l_{a}+l_{b}\right)*x-l_{b}*a}{l_{a}},$$

(16) NF:x<a,b=x.

When *x *>*a*, only SF occurs. When *x *<*a*, only NF occurs. As in the case of forward particle passing, we obtain 3*M *particles corresponding to three fates for *b*.

At each NBP iteration, the internal node thus receives both forward and backward particles, for a total number of 6*M *particles. We then re-estimate the parameters of GMM based on 6*M *particles using the EM algorithm [20], and then randomly sample *M *particles from the estimated GMM for the next NBP iteration.

#### The NBP algorithm

First, we initialize the mean values *μ_i _*of three components of GMMs at internal nodes using Eqs. (11) to (13) from gene expression levels at leaf nodes. The standard deviation *σ_i _*of the GMM is initialized to be the standard deviation of gene expression levels across all leaf nodes for the specific tissue. The prior probabilities or mixing weights *w_i _*of Gaussian components are initialized to be uniform. Second, we draw *M *particles from each GMM, and calculate both forward and backward particles using Eqs. (11) to (16) for each internal node. Third, we re-estimate the mean and standard deviation of the GMM using the EM algorithm based on the 6*M *including 3*M *forward and 3*M *backward particles. Finally, we repeat forward and backward particle passing procedures using *M *particles drawn from the re-estimated GMMs. The parameters of GMM will converge after several NBP iterations *T *[18], where *T *is often around 500 ~ 1000 in practice.

**Figure 5:**
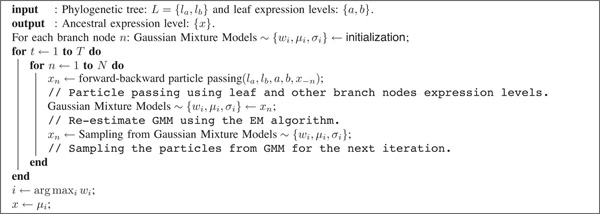
**The NBP algorithm for prediction of ancestral expression levels**.

We estimate the gene expression levels at internal nodes from the GMMs. We choose the mean value of the Gaussian component with the largest mixing weight as the ancestral gene expression level, which is the particle that has the highest likelihood given the GMM. Figure 5 summarizes the proposed NBP algorithm for prediction of ancestral expression levels. Note that the expression level of branch *x_n _*depends on levels of leaf nodes {*a, b*} and other branch nodes *x*_-*n*_.

## Results

**Figure 6:**
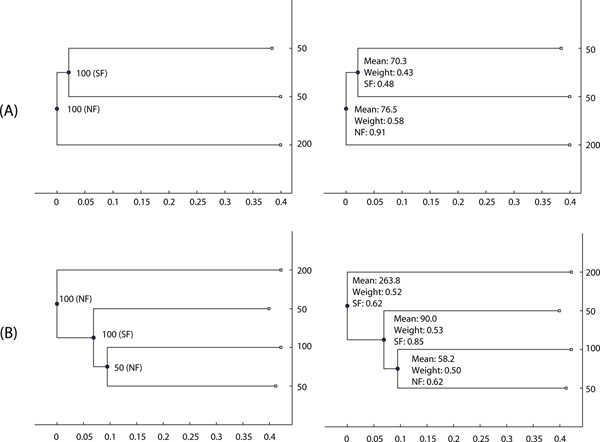
**The ancestral function prediction and SF, CF and NF classification results on two synthetic cases (A) and (B)**. The panel on the left shows synthetic data according to SF, CF and NF rules, where we synthesize the expression levels at internal nodes. The figures on the right shows the prediction results by the NBP algorithm, where "Mean" denotes the mean value of the Gaussian mixture component with the largest mixing weight denoted by the "Weight". We also show the classification probabilities of SF, CF and NF at internal nodes according to Eq. (8), where only the class with the highest probability is shown.

We examined the proposed NBP algorithm and probabilistic model on both synthetic and real data sets. We constructed phylogenetic trees based on 3679 human paralogous families extracted from the Ensembl database [21]. We used the multiple sequence alignment (MSA) algorithm ClustalW2 [22] to align the nucleotide sequences. We built 3679 phylogenetic trees based on the aligned sequences using the TreeBest tools (http://treesoft.sourceforge.net/treebest.shtml). Due to uncertainty, the gene expression level prediction for higher-layer (closer to the root) internal nodes are not robust [19]. In this initial application, we consider two-layer and three-layer phylogenetic trees with topologies depicted in Figure 6. Thus, we obtained 261 two-layer and 148 three-layer phylogenetic trees with leaf nodes having expression levels. Our trees are naturally rooted by virtue of being subtrees of larger paralogous gene trees. In phylogenetic trees, we used the indices 1, 2 and 3 to represent internal nodes from the older to younger ancestors. For accurate parameter estimation, we ran *T *= 1000 iterations of the NBP algorithm in Figure 5. Below we first evaluated the validity of our approach on synthetically generated data where the gene expression levels are generated according to randomly chosen (but known) evolutionary fates of duplicates at each internal node. We then applied our approach to the comprehensive set of human paralogous families to assess relative propensity of their evolutionary fates.

### Synthetic data

We generated a synthetic data set that follows SF, CF and NF rules. For example, the left panel on Figure 6A shows that the expression level of internal node 1 is 100. We randomly assigned a class NF to the internal node 1. According to the NF fate, one duplicate (internal node 2) retains the ancestral function and the other duplicate (the leaf node) develops a new function with expression level 200. For simplicity, we chose twice the ancestral expression as the new expression. Similarly, we randomly assigned a class SF to the internal node 2, which leads to one duplicate having expression level 50 and the other duplicate having expression level 50. For simplicity, we chose half the ancestral expression as the expression of each duplicate.

We first estimated the expression levels at internal nodes 1 and 2 based on the expression levels at leaf nodes. The right panel on Figure 6A shows the prediction results. We chose the mean value of the Gaussian mixture component with the largest mixing weight as the prediction value. We found that the expression value at internal node 2 is predicted as 70.3, and at internal node 1 as 76.5. We normalized the feature point {70.3, 50, 50} and classified it according to Eq. (8). This results in SF having the highest probability 0.48 for the internal node 2. Similarly, NF has the highest probability 0.91 for the internal node 1 based on the normalized feature point {76.5, 70.3, 200}. Thus, our approach faithfully recovers the true classes of the two internal nodes with different probabilities.

Figure 6B shows another example for a three-layer phylogenetic tree. From four expression levels at leaf nodes, NBP infers that the expression level at internal node 1 is 263.8 with a mixing weight 0.52. Thus, this node is classified as SF class with probability 0.62. Also, NBP infers that the expression levels at internal nodes 2 and 3 are 90.0 and 58.2, leading to SF with probability 0.85 and NF with probability 0.62, respectively. Due to more uncertainty at higher-layer internal nodes, the prediction of gene expression level is more inaccurate, resulting in more misclassification cases at internal node 1.

We calculated the classification accuracy of evolutionary fates for internal nodes as

(17) $$\text{Accuracy}=\frac{\text{True Positives}}{\text{Total Number of Internal Nodes}}\times 100\%,$$

where the true positives are the number of internal nodes with correctly predicted class labels. Table 1 summarizes the average classification rate and the standard deviation on five randomly-generated synthetic data sets. All classification rates are much higher than the random guess with 33.3% chance, confirming the effectiveness of the NBP prediction algorithm. Overall, younger ancestors at internal nodes 2 or 3 have much higher classification rates than older ancestor at internal node 1, which is consistent with our intuition that inference for higher-layer nodes (older duplication event) is less robust due to uncertainty. In interpreting the results on the real biological data below, we will place special emphasis on these younger duplicates.

**Table 1:** Classification rates (%) (mean ± std) on five random synthetic data sets

Internal Nodes	1	2	3
Two-layer Trees	43.7 ± 2.1	77.0 ± 1.8	-
Three-layer Trees	38.8 ± 2.4	58.4 ± 1.7	67.2 ± 1.1

### Real data

**Figure 7:**
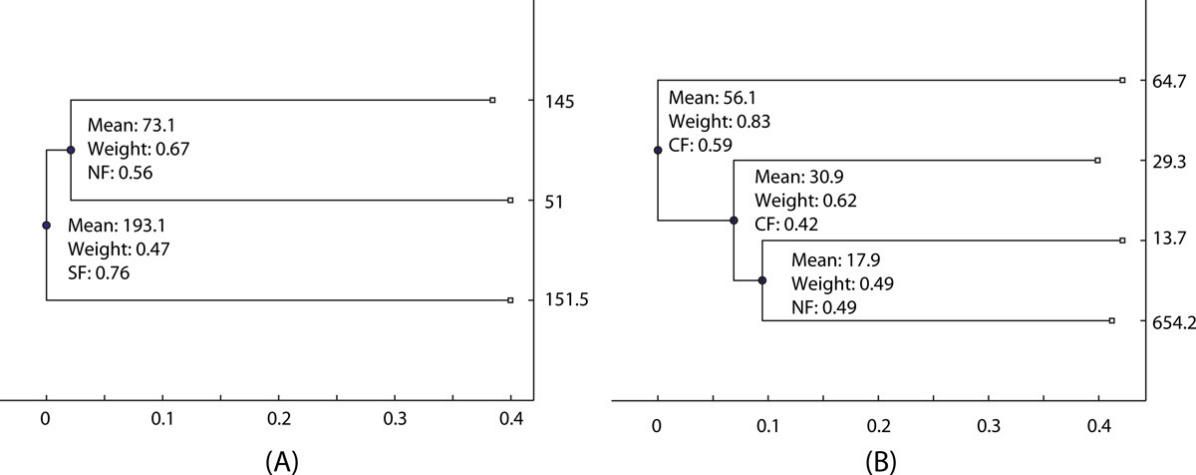
**The prediction results on two trees using real expression levels in Brain Amygdala**.

Human tissue-specific gene expression data for 79 unique tissues were extracted from GEO [23] based on the same Affymatrix array platform. We analyzed each tissue data independently to infer evolutionary fates of gene duplication events. Figure 7 shows two examples for prediction results based on real gene expression levels in Brain Amygdala. For a two-layer tree in Figure 7A, the internal node 2 is classified as NF because the sum of two expression levels at leaf nodes is much more than twice of their ancestral expression level. The internal node 1 is classified as SF because the sum of two expression levels at internal node 2 and one leaf node is close to the predicted expression value. For a three-layer tree in Figure 7B, the internal node 3 is classified as NF because one leaf node has a significantly higher level 654.2. The internal node 2 is classified as CF because the sum of both duplicates is close to twice of its level. Similarly, the internal node 1 is classified as CF due to dosage effects.

We applied the NBP algorithm to 261 two-layer and 148 three-layer phylogenetic trees using gene expression levels of 79 tissues, and summarized the relative propensities of SF, CF and NF among the internal nodes. Tables 2 and 3 show the average proportions of three evolutionary fates across all tissues and all phylogenetic trees at internal nodes 1, 2 and 3, respectively. Because gene expression levels less than 200 can be viewed as noise, we removed those trees with all leaf nodes having less than 200 expression levels. We next focused on high-confidence class predictions by only considering the predicted probability greater than 0.75. Out of a total of 15144 (total number of branch nodes) predictions for two-layer trees, 3250 (22%) and 6350 (45%) are predicted with high-confidence for internal nodes 1 and 2 in Table 2 respectively. Out of a total of 9060 (total number of branch nodes) predictions for three-layer trees, 1723 (19%) and 2244 (26%) and 2621 (27%) are predicted with high-confidence for internal nodes 1, 2 and 3 in Table 3 respectively. These numbers are consistent with greater certainty of inference at lower-layer nodes. Additionally, as a negative control, we also made inferences for scrambled expression data. For each gene, we randomly permuted the gene-expression mapping across different tissues and repeated the experiment. In contrast to real data, a much smaller fraction of permuted cases (7% - 10%) were inferred with high confidence. The results shown in Tables 2 and 3 show that overall there are clear differences in the proportions of evolutionary fates between permuted and real data, and SF is relatively more prevalent at earlier gene duplication event, giving way to CF and NF among recent gene duplication event.

**Table 2:** Proportions (%) for two-layer trees

	SF	CF	NF
Real Overall (node 1)	38.12	34.96	26.92
Real High Confidence (node 1)	53.68	32.27	14.05
Permuted Overall (node 1)	16.59	43.12	40.29

Real Overall (node 2)	16.94	45.35	37.71
Real High Confidence (node 2)	17.93	55.41	26.66
Permuted Overall (node 2)	35.64	35.05	29.31

**Table 3:** Proportions (%) for three-layer trees

	SF	CF	NF
Real Overall (node 1)	30.01	40.59	29.40
Real High Confidence (node 1)	38.14	46.51	15.35
Permuted Overall (node 1)	36.54	39.05	24.41

Real Overall (node 2)	30.47	38.22	31.31
Real High Confidence (node 2)	45.63	37.20	17.16
Permuted Overall (node 2)	21.15	51.80	27.06

Real Overall (node 3)	18.72	43.28	37.99
Real High Confidence (node 3)	18.71	49.40	31.89
Permuted Overall (node 3)	30.62	35.65	33.74

**Figure 8:**
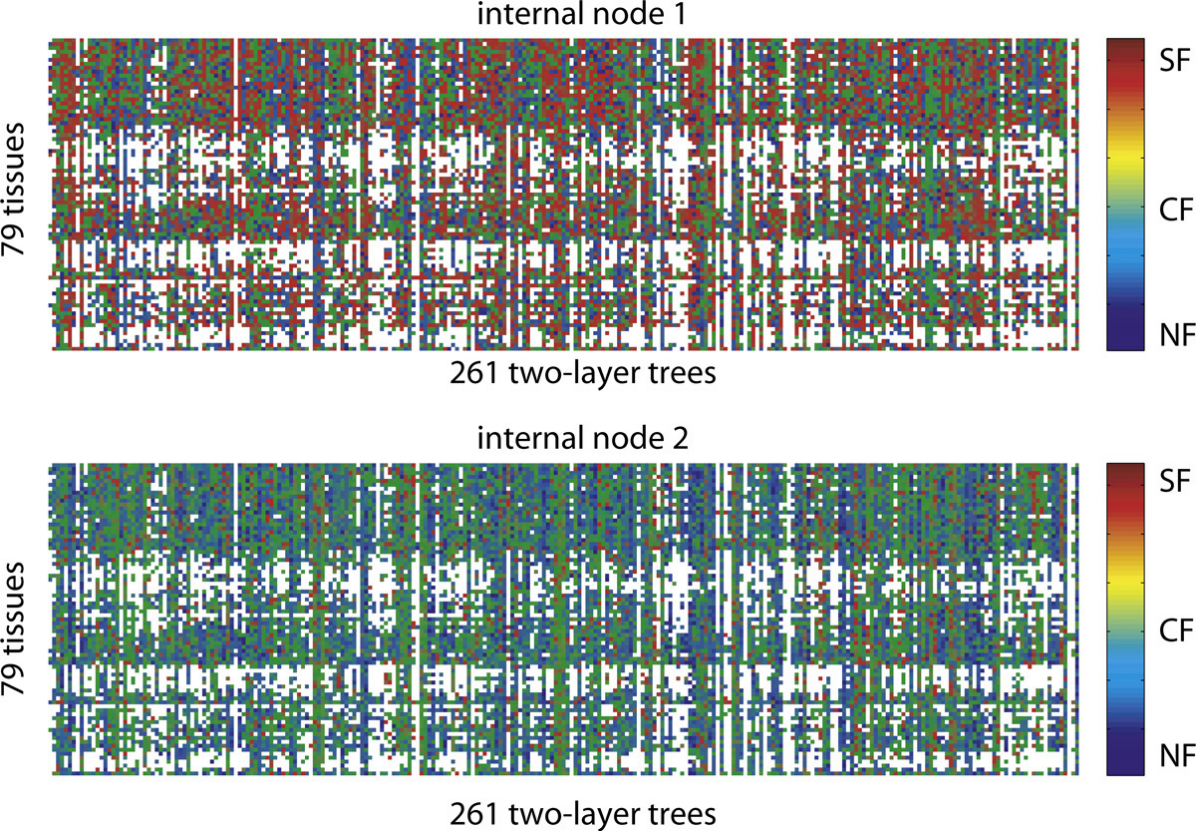
**Three evolutionary fates are represented by RGB colors across 79 tissues (rows) and 261 two-layer trees (columns)**. At each pixel, red denotes SF, green denotes CF and blue denotes NF. The white color denotes those leaf nodes having less than 200 gene expression levels.

Thus far, for each node, we selected the most likely fate, which obscures the relative proportions of the other fates. Figure 8 instead shows the proportions of three evolutionary fates inferred at every node for 79 tissues and 261 two-layer trees. We encoded the three fates by three primary colors - SF (red), CF (green), and NF (blue), *e.g*., [0.2, 0.7, 0.1]^T^, such that the resulting color depicts the proportions. Figure 8 reveals that (1) at node 2 predominant fate is CF and NF while SF is very rare, (2) at node 1, in each tissue, there is substantial variability in the proportion of fates across gene families, and (3) each gene family is dominated by one of the three fates in all tissues. These observations are also supported by the 148 three-layer trees across 79 tissues. Next, we focused on relative proportions of NF and SF.

**Figure 9:**
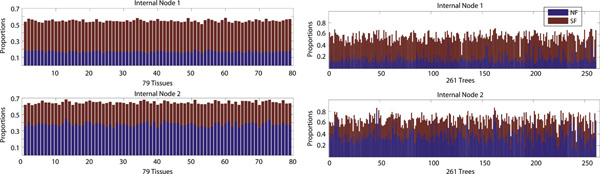
**Left panel: average SF and NF proportions for 79 tissues**. Right panel: average SF and NF proportions for 261 phylogenetic trees. Red denotes SF and blue denotes NF.

Figure 9 (left panel) shows the average SF and NF proportions over 79 tissues, which are the mean values of Figure 8 across 261 trees (columns) without CF. Despite the variability across trees for each tissue (Figure 8), overall SF to NF ratios are similar for all tissues. Moreover, Figure 9 suggests that in relative terms, SF is dominant at internal node 1, and NF is dominant at younger internal node 2. Such tissue-specific evolutionary patterns in three-layer trees are consistent with those of two-layer trees. Figure 9 (right panel) shows the average SF and NF proportions across all tissues for each 261 two-layer trees. Unlike the left panel, the right panel shows quite different SF to NF ratios across different trees, suggesting that the gene evolutionary fates are gene-specific. Although SF at internal node 1 and NF at internal node 2 are dominant in general, in some cases, NF at internal node 1 and SF at internal node 2 are predominant. Such tree-specific evolutionary patterns in three-layer trees are also consistent with those of two-layer trees.

Finally, for each of the three evolutionary fates, we compiled the 50 genes which experienced the particular fate most often. We then tested whether these genes were enriched for certain biological functions using the NIH's DAVID tool [24]. Using the false-discovery-rate cutoff of 10, we found that genes undergoing SF were enriched for chromosome organization and RNA binding, genes undergoing CF were enriched for intracellular signaling cascade and homeostatic process, and genes undergoing NF were enriched for transcription regulator activity and transcription factor activity.

## Conclusion and discussion

In this paper, we have introduced a novel integrative strategy for two important and interrelated evolutionary inference problems--those of gene expression and of strategy to measure the proportions of three evolutionary fates (SF, CF and NF) of gene duplicates. Our main contribution is the development of a probabilistic framework for the dual inference problem, which includes: (1) a novel description of SF, CF and NF with respect to tissue-specific gene expression levels, (2) the NBP algorithm to predict the ancestral gene expression level, which explicitly incorporates the distinct evolutionary fates at internal nodes, and (3) a probabilistic model to classify SF, CF and NF fates. While our formulation of evolutionary fates in terms ancestral and descendent expression level is reasonable, it does not account for all possible configurations of (*x, a, b*), for instance, *a, b *< <*x*, which would correspond to gene expression loss. There are six possible configurations of (*x, a, b*) without differentiating between *a *and *b*. It may be worthwhile modeling all these configurations in future extensions of this work. We are currently pursuing this as future work.

Comprehensive applications to human tissue-specific expression data suggest that SF is more prevalent during the early phase of gene family expansion, giving way to NF at a later phase of gene family expansion. Excluding CF, which is common to both early and late phase of gene family expansion, we refer to this order of events as "SF-NF". Our "SF-NF" model is consistent with some previous studies. Wagner and colleagues [25] analyzed Saccharomyces cerevisiae PPI data and compared the age of paralogs to the number of shared interaction partners. They found that most older duplicates lose on average 80% ~ 90% shared partners. The rapid loss of common interacting partners between duplicates suggests that SF occurs in earlier duplicates. He and Zhang [6] also analyzed Saccharomyces cerevisiae PPI data sets and human tissue expression profile data (not tissue-specific expression level as we do). They found that the number of shared partners decreases rapidly and then the number of unique interactions increases slowly with the age of duplicates. They speculated that most duplicates first experience a rapid SF followed by a prolonged period of NF. Although [7] questioned the prevalence of NF among younger duplicates from many aspects of the PPI data set, the proposed "SF-NF" hypothesis is based on a different strategy and human tissue-specific gene expression data that avoid the pitfalls in the PPI data set. VanderSluis and colleagues [12] analyzed the genetic interactions in Saccharomyces cerevisiae and argued that SF is the major driving force behind duplicate gene retention. They showed that one duplicate degrades faster than the other and often becomes functionally specialized.

Our model can capture pure SF or NF scenarios. For instance, if the highest-layer internal node has the largest expression value, then all leaf nodes split this expression value thus inferring pure SF. By contrast, if the highest-layer internal node has the smallest expression value, then all leaf nodes increase in the expression value leading to pure NF inference. However, our results suggest that the pure SF or NF hypothesis is unlikely. Using human tissue-specific gene expression data, the NBP algorithm, and the probabilistic model (Figure 3), our results suggest that the most probable hypothesis is "SF-NF". Unlike previous studies, we find CF is also a major mechanism in functional divergence in both older and younger duplication events. A significantly lower proportion of SF in younger duplicates suggests a decrease in specialization at a later phase of gene family expansion, giving way to an increase in novel function.

Our analysis suggests that genes belonging to different functional categories may have different propensities for the various evolutionary fates after duplication. We found that genes involved in homeostasis and intracellular signaling have a greater propensity for CF. SF is more common among genes involved in chromosome organization and RNA binding, which tend to be ancient genes and incidentally SF is more common among earlier duplication events. Similarly NF is more common in transcription factors that have undergone several recent expansions in family size, and incidentally NF is more common among recent duplication events. However, previously it has been argued that there may not be bias among these fates in terms of functional categories, and dosage compensation is likely to be a major force for gene retention in the beginning and once retained genes are likely to undergo SF or NF later on [26]. Further interpretations of these findings will require further investigation.

In the current work, we do not distinguish duplication on two scales: the whole genome duplication (WGD) and small-scale duplications (SSD) [27]. Our approach can be applied to expression data of some organisms having undergone recent whole genome duplication like Arabidopsis, which may reveal different evolutionary fates between WGD and SSD duplicates.

In summary, our work presents the first integrative probabilistic framework for jointly inferring gene expression evolution and inferring evolutionary fates of gene duplicates using tissue-specific gene expression as a proxy for gene function. In doing so, our work addresses conceptual pitfalls of previous frameworks to study these problems and we believe that this work will serve as guide for future works in this area. Based on synthetic data we have shown our method to be reasonably accurate, especially with regards to recent duplicates. Our comprehensive application to human gene duplicates also provides novel insights. Although the specifics of results on biological data should be taken with a grain of salt because the tissue-specific expression data has inherent variability as well as technology-dependent noise in gene expression measurements, it is encouraging that general patterns are consistent between two-level and three-level tree analyses. Notwithstanding the inherent difficulties in verifying the biological results, the main merit of this work lies in the development of a new principled framework for the dual evolutionary inference problem, and its verification using simulation. As RNA-seq is systematically applied to a wider array of tissues, the methods developed here can be applied to gain further insights into gene expression evolution and fates of gene duplicates.

## Authors' contributions

JZ conceived this idea, carried out experiments, and wrote this paper. SH refined this idea, revised this paper, and initiated this project.

## Competing interests

The authors declare that there are no competing interests.

## Declarations

The publication of this article is supported by NSFC (Grant No. 61003154), the Shanghai Key Laboratory of Intelligent Information Processing, China (Grant No. IIPL-2010-009), Natural Science Foundation of the Jiangsu Higher Education Institutions of China (Grant No. 12KJA520004) to JZ and NIH grant R01GM085226 to SH.

This article has been published as part of *BMC Genomics *Volume 14 Supplement 1, 2013: Selected articles from the Eleventh Asia Pacific Bioinformatics Conference (APBC 2013): Genomics. The full contents of the supplement are available online at http://www.biomedcentral.com/bmcgenomics/supplements/14/S1.
